# Door to the cell for COVID-19 opened, leading way to therapies

**DOI:** 10.1038/s41392-020-00215-6

**Published:** 2020-06-26

**Authors:** Zhiwei Huang, Jijie Chai

**Affiliations:** 1 Center for Life Sciences, School of Life Science and Technology, Harbin Institute of Technology, Harbin, 150080 China; 2 School of Life Sciences, Tsinghua University, Beijing, 100084 China

**Keywords:** Structural biology, Structural biology

**A very recent study by Lan et al.**^1^**published in*Nature*determined the crystal structure of the severe acute respiratory syndrome coronavirus (SARS-CoV)-2 receptor-binding domain (RBD) bound to angiotensin-converting enzyme 2 (ACE2). The structure reveals the mechanism of SARS-CoV-2 RBD recognition by its receptor ACE2, which is highly conserved in ACE2 recognition of SARS-CoV RBD. The study provides structural information on developing small molecules targeting SARS-CoV-2 RBD/ACE2 and implies the existence of other mechanisms than receptor binding for the markedly different infection activity of the two evolutionarily close viruses.**

The outbreak of a novel and highly pathogenic coronavirus (SARS-CoV-2) has presented a serious global public health emergency of coronavirus disease 2019. As of 11 May 2020, more than 4 million cases have been confirmed with the infection, leading to nearly 279,000 deaths in 214 countries (https://www.who.int), and the coronavirus continues to spread quickly all over the world. Currently, effective vaccines or antiviral drugs for SARS-CoV-2 are unavailable. The newly identified SARS-CoV-2 belongs to β-coronavirus, which also includes Middle East respiratory syndrome coronavirus (MERS-CoV) and SARS-CoV. The spike glycoprotein of coronaviruses acts as an important determinant of their virulence activity by interacting with a receptor on the surface of host cells.^2^ The interaction between the spike glycoprotein and its receptor can serve as a target for therapeutic interventions to treat diseases caused by coronaviruses. ACE2 has been identified as a functional receptor of SARS-CoV.^3^ More recently, ACE2 was also shown to be a receptor of SARS-CoV-2.^4^ The ectodomain of the spike protein contains a receptor-binding unit S1 and a membrane-fusion unit S2. Interaction of the RBD from the S1 unit with ACE2 leads to fusion of S2 with the host cell and viral membranes,^2^ thus mediating entry of coronavirus into host cells.

**Fig. 1:**
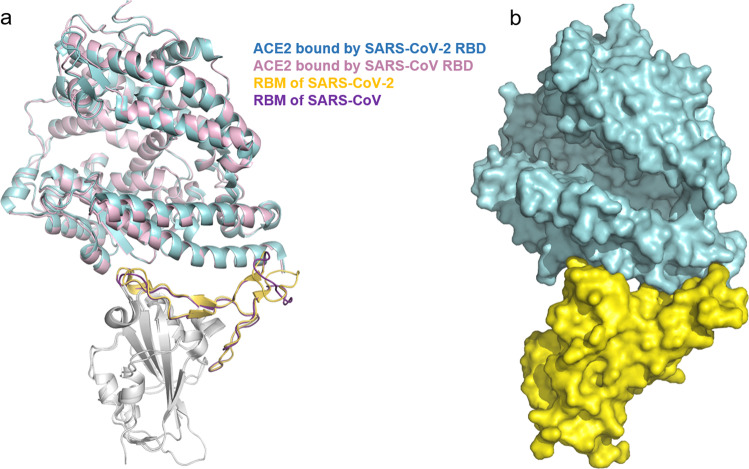
Structural superposition of SARS-CoV-2 and SARS-CoV RBMs binding to ACE2 receptor. Structural alignment of the SARS-CoV-2 RBD/ACE2 (cyan) and SARS-CoV RBD/ACE2 (pink) complexes (left). The RBM of SARS-CoV-2 and SARS-CoV is shown in yellow and purple, respectively. The remaining portion of the two RBDs is shown in gray. The PDB codes for SARS-CoV-2 RBM/ACE2 and SARS-CoV RBM/ACE2 are 6M0J and 2AJF, respectively. The interface of SARS-CoV-2 RBD and ACE2 receptor is shown in surface view (right). RBD and ACE2 are colored in yellow and cyan, respectively

To elucidate the mechanism of SARS-CoV-2 RBD and ACE2 interaction, Lan et al.^1^ determined the complex structure of the two proteins at 2.45 Å resolution by X-ray crystallography. The final structural model contains residues of Thr333-Gly526 of the SARS-CoV-2 RBD, and residues of Ser19-Asp615 of the ACE2 N-terminal peptidase domain (Fig. 1). The receptor-binding motif (RBM) at one side of SARS-CoV-2 RBD forms a concave for interaction with ACE2. The overall structure of the SARS-CoV-2 RBD is highly similar to that of the SARS-CoV RBD.^2^ This is not surprising given 72% sequence identity of the two RBDs. However, remarkable conformational differences occur to the loop from the distal end of the RBM that faces toward the solvent region. Structural comparison showed that the ACE2-bound SARS-CoV-2 RBD is nearly identical with that from the free SARS-CoV-2 spike protein, indicating that ACE2 binding induces no notable conformational changes in SARS-CoV-2 RBD.

Specific recognition of ACE2 by SARS-CoV-2 involves two β-sheets (β5 and β6) and three connecting loops of the RBM. Structural superimposition showed that SARS-CoV-2 RBD and SARS-CoV RBD employ a highly conserved mechanism for interaction with ACE2, supporting a close evolutionary relationship between the two viruses. Both of the RBM-ACE2 interfaces feature a large network of hydrogen bonds, highlighting specific interaction between the two proteins. Fourteen of the ACE2-interacting residues are shared by the two RBDs, of which 8 are identical and 5 are similar. The shared but non-conserved residue interacts with the same set of amino acids of ACE2. The conserved interactions in the SARS-CoV-2 RBM/ACE2 and SARS-CoV RBM/ACE2 complexes suggests that the two RBDs can have a similar affinity with ACE2. Indeed, quantification assays using surface plasmon resonance showed that SARS-CoV-2 RBD and SARS-CoV RBD bound to the ACE2 receptor with an affinity ~4.7 and ~31.0 nM, respectively. One notable difference between two complex structures is that Lys417 of SARS-CoV-2 is located outside the RBM but forms salt-bridge interactions with Asp30 of ACE2. By comparison, the equivalent position of SARS-CoV has a valine residue, which is unlikely to form the salt bridges seen in the SARS-CoV-2 RBD/ACE2 complex. This subtle structural difference was proposed to contribute to the slightly higher affinity between SARS-CoV-2 RBD and ACE2. However, ~20-fold difference between SARS-CoV-2 (Kd of 14.7 nM for ACE2) and SARS-CoV (Kd of 325 nM for ACE2) in binding affinity with ACE2 was observed by another group,^4^ probably due to different proteins used in the assays. The study by Lan et al.^1^ also provided an explanation for the observation that none of the isolated SARS-CoV monoclonal antibodies are able to neutralize SARS-CoV-2. By mapping the epitope residues of SARS-CoV RBD onto the sequence of SARS-CoV-2 RBD, they found that one third (7 over 21) epitope positions of the antibody 80R are altered in SARS-CoV-2 RBD. A similar observation was also made with the epitope positions of the antibody m396.

Altogether, the study by Lan et al.^1^ revealed the structural mechanism of ACE2 receptor recognition by SARS-CoV-2 RBD. The mechanism sheds light on pathogenesis of the highly pathogenic virus and can serve as a template for developing intervention strategies targeting SARS-CoV-2 spike and receptor recognition. It is unexpected that SARS-CoV-2 and SARS-CoV employ a nearly identical mechanism for interaction of the ACE2 receptor, given their striking differences in infection and transmission activity.^5^ The mechanism underlying the differences remains poorly understood, but the small difference between the two viruses in affinity with ACE2 is less likely to determine their distinct infection and transmission activity. As proposed by the authors, some unique SARS-CoV-2-encoded proteins might have an important role in this aspect. It also remains possible that SARS-CoV-2 has other receptors(s) than ACE2 for entry into the host cells. Addressing this question warrants future studies and will be conducive to developing antiviral therapies.
